# Electrophysiological modulation of sensory and attentional processes during mind wandering in attention-deficit/hyperactivity disorder

**DOI:** 10.1016/j.nicl.2020.102547

**Published:** 2020-12-30

**Authors:** Natali Bozhilova, Jonna Kuntsi, Katya Rubia, Giorgia Michelini, Philip Asherson

**Affiliations:** a Social, Genetic and Developmental Psychiatry Centre, Institute of Psychiatry, Psychology and Neuroscience, King’s College London, De Crespigny Park, London SE5 8AF, United Kingdom; b Semel Institute for Neuroscience & Human Behavior, University of California Los Angeles, 760 Westwood Plaza, Los Angeles, CA 90024, United States; c Department of Child and Adolescent Psychiatry, Institute of Psychiatry, Psychology and Neuroscience, King’s College University London, De Crespigny Park, London SE5 8AF, United Kingdom

**Keywords:** ADHD, Mind wandering, Perceptual decoupling, Attention allocation, Event-related potentials

## Abstract

We recently reported increased mind wandering (MW) frequency in adults with attention-deficit/hyperactivity disorder (ADHD) relative to controls during high demands on sustained attention, reflecting deficient context regulation of MW. Studies on community samples previously linked context regulation of MW with attenuation in brain sensory processes, reflecting perceptual decoupling, and attentional processes during MW compared to task focus. However, the association between deficient context regulation of MW and these neural processes has not been studied in ADHD. We addressed this question by comparing adults with ADHD (N = 23) and controls (N = 25) on event-related potentials of early sensory processes (P1) and attention allocation (P3) during tasks manipulating cognitive demands (high vs low) on working memory and sustained attention, and during periods of MW and task focus measured through experience-sampling. Compared to controls, adults with ADHD showed reduced P1 during high sustained attention demands, as well as reduced P3 during high working memory demands. These group differences were no longer significant after adding MW frequency as a covariate. Across tasks, adults with ADHD showed no differences from controls on the P1 during MW episodes, but attenuated P1 during task focus. P3 was reduced in adults with ADHD compared to controls during MW, but not during task focus during the sustained attention task. These findings converge to indicate that impairments in early sensory processing in individuals with ADHD seem parallel to increased MW frequency and might reflect inefficient adjustments from periods of MW to task focus.

## Highlights

• Adults with ADHD relative to controls reported lower P1 during high demands on sustained attention.
• Adults with ADHD also showed lower P1 during task focus, but not during mind wandering than controls.
• Increased mind wandering frequency in ADHD might account for these between-group effects.

## 1 Introduction

Attention-Deficit/Hyperactivity Disorder (ADHD) is a neurodevelopmental disorder affecting 5–7% of children (Polanczyk et al., 2014) and 2–3% of adults worldwide (Fayyad et al., 2017). Adults with ADHD often describe excessive frequent and spontaneous mind wandering (MW; Asherson, 2005), reflecting an uncontrolled drift of attention away from the primary task (Franklin et al., 2017, Seli et al., 2015). Experience-sampling for periods of MW and self-report MW scales in adults (Seli et al., 2015; Franklin et al., 2017, Mowlem et al., 2016, Mowlem et al., 2019, Biederman et al., 2019) and children with ADHD (Van den Driessche et al., 2017, Frick et al., 2020) further show that MW is increased in ADHD across the lifespan. Based on these observations, we developed a new MW perspective on ADHD which proposed that MW is a core process in ADHD and that deficient regulation of neural activity underlying MW might explain the inattentive symptoms and cognitive performance deficits seen in ADHD (Bozhilova et al., 2018).

Key characteristics of MW are context regulation and perceptual decoupling. Context regulation is the term used to describe a decrease in MW frequency as task demands increase to preserve task performance (Smallwood and Andrews-Hanna, 2013); indicating adaptation of MW frequency to task demands. This was first described in a study by Smallwood and colleagues who found increased MW frequency during a low demand (0-back) condition compared to a high demand (1-back) condition of a sustained attention task (Smallwood et al., 2009). Context regulation of neural activity to changing task demands may also be observed, for example in the deactivation of default mode network activity to increasing demands on sustained attention in healthy controls (Christakou et al. 2013). Studies on college students and community samples have shown context regulation of both MW frequency (Forster and Lavie, 2009, Levinson et al., 2012, McVay and Kane, 2012, Xu and Metcalfe, 2016, Ruby et al., 2013, Smallwood et al., 2007) and neural activity (e.g. reduced activity, or deactivation of the default mode network as task demands increase; Mason et al., 2007).

Numerous previous studies have shown deficient context regulation of neural activity in ADHD compared to controls (Skirrow et al., 2015, Rommel et al., 2016, Christakou et al., 2013, Liddle et al., 2011, Bollmann et al., 2017). We therefore hypothesised that such deficient neural adaptation to task demands may underlie a deficit in ADHD in the context regulation of MW frequency (e.g., a lack of decrease of MW frequency during high demand conditions; Bozhilova et al. 2018). In support of this view, using the same sample as in the current study, we recently provided behavioural evidence of deficient context regulation of MW during increasing demands on sustained attention, but not on working memory, in adults with ADHD (Bozhilova et al., 2020b). Controls were able to maintain consistently low MW frequency in response to task demands, while adults with ADHD showed consistently high MW frequency during the sustained attention task. No previous study, however, has tested whether the neural processes underlying the regulation of MW during different task conditions are deficient in ADHD. Here, we investigate the neural underpinnings of MW in individuals with and without ADHD using the millisecond resolution of event-related potentials (ERPs).

Another feature of MW is perceptual decoupling (Schooler et al., 2011). This refers to a switch from processing external sensory stimuli to processing internal thoughts and attenuated attention to external sensory stimuli during periods of MW compared to on-task focus (Smallwood and Schooler, 2015). Of relevance to perceptual decoupling and context regulation is the P1, an ERP which reflects a pre-attentive and automatic sensory response trigged by visual stimuli within the 100 ms of stimuli presentation (Luck, 2005). P1 has consistently been shown to be attenuated during episodes of MW as opposed to task focus (referred to as perceptual decoupling) (Kam et al., 2011, Baird et al., 2014, Broadway et al., 2015, Kam and Handy, 2014, Martinon et al., 2019). P1 is also closely associated with impaired cognitive performance during cognitive control and attentional tasks, including increased mean reaction time (MRT), reaction time variability (RTV) and error rate (Smallwood et al., 2004, Smallwood and Schooler, 2006; McVay and Kane, 2009, Stawarczyk et al., 2011, Kam et al., 2012).

The role of early sensory processing and perceptual decoupling remain poorly understood in ADHD, as only a few studies have examined P1 components in ADHD samples. Available studies have reported attenuated P1 in individuals with ADHD compared to controls (Dockstader et al., 2008, Nazari et al., 2010, Gohil et al., 2017), but some have found no group differences (Kim et al., 2014, Hasler et al., 2016). In addition, P1 has been shown to increase following methylphenidate treatment in individuals with ADHD (Lee et al., 2005). In the absence of performance differences, comparable P1 amplitudes have been interpreted as a compensatory mechanism in individuals with ADHD (Kóbor et al., 2015, Shahaf et al., 2012). Since population-based studies suggest that P1 varies as a function of MW, it is possible that P1 reductions in individuals with ADHD are a neural marker of increased MW in ADHD. However, no previous study has examined whether P1 components in the context of MW versus task focus in ADHD samples.

Another ERP component relevant to context regulation is the P3, which arises 250–300 ms after stimulus presentation and broadly reflects attention allocation (although its interpretation depends on the task conditions) (Polich, 2007). P3 attenuations have consistently been reported during MW episodes in population-based samples (Barron et al., 2011, Riby et al., 2008, Smallwood et al., 2008, Villena-González et al., 2016, Smallwood, 2013). Meta-analyses also indicate that reduced P3 during cognitive control and attentional tasks is one of the most replicated ERP findings in ADHD (Szuromi et al., 2011, Kaiser et al., 2020). In individuals with ADHD, attenuated P3 has also been associated with increased RTV, and an increase in P3 from a slow, baseline to a fast-paced, rewarded condition was associated with a decrease in RTV between the two conditions (Cheung et al., 2017). These findings suggest parallel modulations in P3 amplitude and response variability with changing task demands. Further, we recently reported that reduced P3 during the Sustained Attention to Response Task (SART) is strongly associated with both ADHD and self-reported MW (Bozhilova et al., 2020a). However, P3 has yet to be studied in relation to periods of MW and task focus in individuals with ADHD, and it thus remains unclear whether P3 reductions underlie periods of MW in ADHD samples.

Taken together, the studies highlight reductions in both P1 and P3 during MW episodes in community samples, and during cognitive tasks in ADHD samples, but these neural processes have yet to be studied in the context of MW in ADHD. This study aimed to address this gap in the literature by investigating the association between context regulation of MW and ERPs of early sensory processing (P1) and attention allocation (P3) in adults with and without ADHD through two complementary analyses. Firstly, we examined differences between groups on P1 and P3, as well as within-group modulations of these ERPs with changing task demands, and whether these effects were explained by MW frequency (Analysis 1). We predicted that P1 and P3 in individuals with ADHD would be reduced compared to controls across tasks. With regard to within-group effects, we predicted a within-group decrease in P1/P3 from high to low demands on working memory (1-back vs 0-back) and with longer delays between visual stimuli (from low to high, 2 s vs 5 s vs 8 s) in the ADHD group, but comparable ERPs across task conditions in controls. We also expected a within-group increase in sensory processing (P1) during the shortest and most frequent delays (1 s) in controls, but not in individuals with ADHD, as these delays place high demand on sensorimotor function (Christakou et al., 2013). Based on our previous findings showing greater MW frequency in adults with ADHD compared to controls during these tasks (Bozhilova et al., 2020b), we further hypothesized that MW frequency would statistically account for these between- and within-group effects. Secondly, to further evaluate the relationship of MW with cognitive and neural deficits in ADHD, we examined the effect of MW on ERP and performance measures by contrasting periods of MW and task focus in the ADHD and control groups (Analysis 2). Based on previous literature (Bozhilova et al., 2018), we predicted decreased P1/P3 and worse performance during periods of MW in the ADHD group compared to controls, but comparable ERPs and performance between groups during task focus.

## 2 Method

Twenty-three participants with ADHD and 25 controls were included. This sample met quality control criteria for electroencephalography (EEG) data (see *EEG analyses/data screening* below for details), from an original sample of 27 adults with ADHD and 29 controls. Adults with ADHD were recruited from the South London and Maudsley NHS Trust and Barnet, Enfield and Haringey Mental Health Trust adult ADHD clinics and online advertisements via adult ADHD networks and primary care physicians. Age-matched controls with low levels of ADHD symptoms (i.e., one or less ADHD symptoms based on diagnostic assessments for this study), and no prior diagnosis or treatment for any mental health condition were recruited via online recruitment advertisements from all over London. Participants in both groups were excluded if they reported a current or past diagnosis of major physical illness (e.g. neurological problems, head injury), severe recurrent mental health problems other than ADHD (e.g. psychosis, schizophrenia, bipolar disorder, antisocial personality disorder), current or past substance abuse (defined as more than 8 units of alcohol for males or 6 units for females of alcohol consumed daily, or recreational drug use more than twice weekly), or an IQ < 80.

All ADHD participants had a formal diagnosis of ADHD based on clinical records and met both DSM-IV and DSM-5 criteria for ADHD based on assessments with the Diagnostic Interview for ADHD (DIVA 2.0, Kooij, 2012). Among participants in the ADHD group, twelve were on stable treatment with stimulant medication and two on atomoxetine. Seven participants with ADHD were taking a low dose of a concomitant medication for anxiety or depression. The two groups did not differ on age, sex and IQ (Table 1).

**Table 1:** Comparisons between ADHD and control group on demographic characteristics.

	ADHD (N = 23)	Controls (N = 25)	Group comparison	
	Mean ± SD	Mean ± SD	d	p
Age (years)	36.73 ± 8.67	31.80 ± 11.42	0.47	0.113
IQ	111.50 ± 13.25	114.28 ± 16.72	0.18	0.528
MW frequency	0.57 ± 0.22	0.15 ± 0.14	2.16	0.001*

	Males: Females	Males: Females	Chi^2^	p
Gender	13:10	12:13	0.47	0.521

Abbreviations: ADHD-Attention-deficit/hyperactivity disorder, IQ-Intelligent Quotient from the Wechsler Abbreviated Scale of Intelligence, WASI-II.

Notes: The total MW frequency was calculated using the total number of MW episodes across tasks divided by the total number of all episodes (task focus and MW). *p < 0.05.

### 2.1 Procedure

All participants attended a 3–4 h test session and completed a diagnostic interview for ADHD, cognitive tasks with simultaneous EEG recordings preceded by a 1–2 min practice session for each task, IQ testing and self-report questionnaires. Participants were asked to refrain from consuming caffeine, alcohol, illicit and non-illicit substances or smoking on the day of the assessment. Participants with ADHD were also asked to stop taking stimulant medication for 48 h before the assessment, as is standard practice in ERP studies of ADHD samples (Michelini et al., 2016, Wiersema et al., 2009, Fisher et al., 2011, Marquardt et al., 2018). On the day of the assessments, all participants confirmed that had successfully followed these instructions. Withdrawal effects are unlikely to have impacted the study findings. Since such effects are not a feature of the therapeutic use of stimulant medications for ADHD and controlling for dose or period of time on medication is rarely, if ever, included in ADHD studies.

### 2.2 Cognitive tasks

#### 2.2.1 Mind wandering task

**Fig 1:**
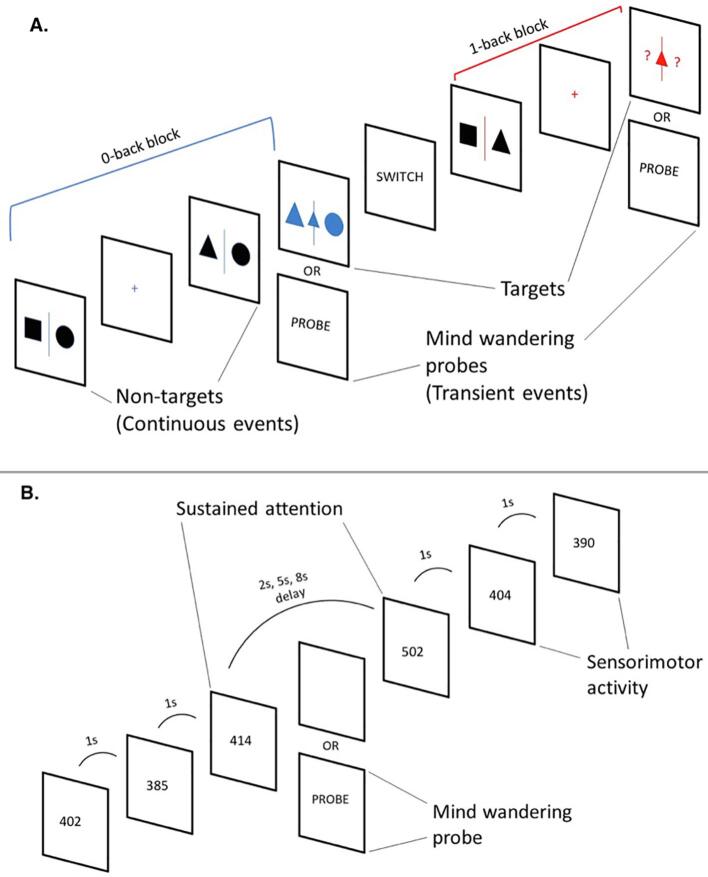
Schematic representation of the cognitive tasks. A. Mind Wandering Task: The Participants alternated between the two conditions. One condition involved observing two black shapes (non-target) before three blue shapes (target) appeared. At that point, the participant had to indicate which of the two side shapes matches the small blue shape in the middle (choice reaction, 0-back). In the 1-back condition, participants had to encode in working memory the two black shapes and when a small red shape with two red question marks on each side appears, they had to choose the left or right question mark based on the position of the black shape that is identical to the small red shape in the prior trial (working memory, 1-back) (Konishi et al., 2015). B. Sustained Attention task: The participants were asked to respond as fast as possible to the appearance of black-counters (participant’s reaction time) on the screen that count up in milliseconds. The counters appeared either after frequent and predictable delays of 1 s in blocks of 3–5 stimuli, or after unpredictable long delays of 2, 5 or 8 s, pseudo-randomly interspersed into the blocks of 1 s delays (Christakou et al., 2013). (For interpretation of the references to colour in this figure legend, the reader is referred to the web version of this article.)

The 0-back (choice reaction) condition measures general alertness and motor speed, whereas the 1-back condition measures visual working memory performance (Konishi et al., 2015). In the 0-back condition, participants observed a sequence of black shapes (separated with a blue line into a right and a left shape) in the middle of the computer screen while waiting for a blue target (a small shape with two bigger shapes on each side). Upon target presentation, they had to indicate the location of the bigger shape which matched the small target shape by pressing the left or the right arrow. In the 1-back condition, participants were exposed to the same sequence of black shapes (separated by a red line into a right and a left shape) and were intermittently presented with two red question marks (‘?’) with a small red shape (target) between the question marks. When the question marks appeared, the participants had to make a manual response to indicate the location (left or right) of the shape in the previous trial that was identical to the small target shape. Because the occurrence of the colored question marks was randomly determined, this task required participants to encode and retain in memory the location (left or right) of each non-colored shape (Fig. 1).

The order of conditions was counterbalanced. For each trial, between 2 and 6 non-targets preceded the target. The non-targets lasted for 1 to 3 s with increasing steps of 0.1 s in each trial (the maximum interval length was 3 s for each trial). The total number of stimuli was 128 targets (64 in each condition) and 580 non-targets (290 in each condition). Each target lasted for 4 s, allowing the participant 4 s to respond until their response ended it immediately. The fixation appeared before and after all task stimuli crosses ranged from 2 to 4 s with increasing steps of 0.1 s.

There was a total of 8 trials in each block for each condition. There were 8 blocks, with a varying duration from 40 s to 120 s. At the end of each block, participants were informed that they were about to start a new block with either the same condition with the word “STAY” or that they were about to switch to the other condition with the word “SWITCH”. Both message words “SWITCH” and “STAY” appeared on the screen for 5 s. The total duration of task was approximately 30 min divided into two 15-min sessions.

#### 2.2.2 Sustained attention task (SAT)

The task is a modified version of the SAT (Christakou et al., 2013). The SAT is a vigilance task, which has 3 levels of a progressively increasing sustained attention load (2 s, 5 s, 8 s) (Fig. 1). The participants are required to respond as quickly as possible to the appearance of a counter (i.e. black digits) of milliseconds, via a right button response within 1 s. The visual stimuli appeared either after short, frequent consecutive intervals of 1 s, in series of 3 to 5 stimuli (520 in total, 260 in each session), or after longer, less frequent and unexpected time delays of 2, 5 or 8 s (52 in total, 26 each in each session), pseudo-randomly interspersed into the blocks of 3 to 5 trials of 1 s. The long, infrequent delays place a higher load on sustained attention/vigilance, whereas the short, frequent 1-s delays are typically anticipated and place higher demand on sensorimotor synchronization (Christakou et al., 2013). The total duration of the task was approximately 30 min divided into two 15-min sessions (Fig. 1).

#### 2.2.3 MW Probes

MW was recorded using an experience-sampling approach with thought probes (15 per session, 30 in total) at approximately 1-minute intervals. The probe appeared in the place of the targets in the MWT and in the place of the stimulus following the infrequent delays (i.e., 2 s, 5 s, 8 s) in the SAT. We included 26 delays per session (78 in total) contrasting 20 delays (60 in total) in the original version of the SAT (Christakou et al., 2013). Most of these extra delays (36 in total) were followed by thought probes (30 in total) rather than the task stimulus (black digits), ensuring consistency in the number of delays between our and the original version of the SAT. Participants were first asked “*Where was your attention just before this probe?*” with two response options “*On task*” and “*Off task*”. If they had responded “*Off task*”, another question enquired “*Were you aware of your attention drifting away from the task?”* with two responses options *“Aware”* and “*Unaware”.* MW and task focus episodes were measured in the 15 s preceding each probe, consistent with previous work (Baird et al., 2014; Braboszcz and Delorme, 2011, Kirschner et al., 2012).

#### 2.2.4 Task performance

Task performance measures and MW frequency during each task condition have been reported previously (Bozhilova et al., 2020b) and are summarized in Supplementary Table S1. The current study investigated MRT, RTV and error rate (incorrect responses in the MWT, omission errors in the SAT) in the 15-s period before MW probes as the average value for each MW and task focus episode.

### 2.3 EEG recoding and pre-processing

The EEG was recorded from a 62-channel DC-coupled recording system (extended 10–20 montage) (Brain Products, Gilching, Germany), using a 500 Hz sampling rate, impedances under 10 kΩ, and FCz as the recording reference. The electro-oculograms were recorded from electrodes above and below the left eye and at the outer canthi. The EEG data were analysed using EEGLAB (Delorme and Makeig, 2004). The raw EEG data were downsampled to 256 Hz, re-referenced to the average of all electrodes (turning FCz into an active channel), and digitally filtered using basic Finite impulse response (FIR) filters below 1 Hz and above 30 Hz. Prior to re-referencing, flat channels and channels with extremely large artefacts were removed and replaced with topographic spline interpolation. Sections of data >200 μV were automatically rejected. Ocular artefacts (blinks and lateral eye movements), clearly isolated heartbeat, line noise and muscle artefacts were identified using independent component analysis (ICA) with the Adaptive Mixture ICA (AMICA) algorithm (Palmer et al., 2011). ICA allows for the correction of artefactual data through removal of the artefactual components and back-projection of all but those components. Following the back-projection, all datasets were also visually inspected and sections of data containing residual artefacts were removed manually.

### 2.4 ERP analyses

For Analysis 1, average ERPs were created separately for the working memory (1-back), choice reaction (0-back) and delay (1 s, 2 s, 5 s, 8 s) conditions. The number of trials in each ERP by group are given in Supplementary Table S2. The trials in the 15 s preceding the MW/task focus probes were excluded from Analysis 1 to allow comparability with previous studies using these tasks that did not include thought probes, and because the 15 s period preceding probes was the focus of Analysis 2. For Analysis 2, ERPs were generated in trials included in the 15 s preceding the probe and split between task focus and MW (Supplementary Table S2).

**Fig. 2:**
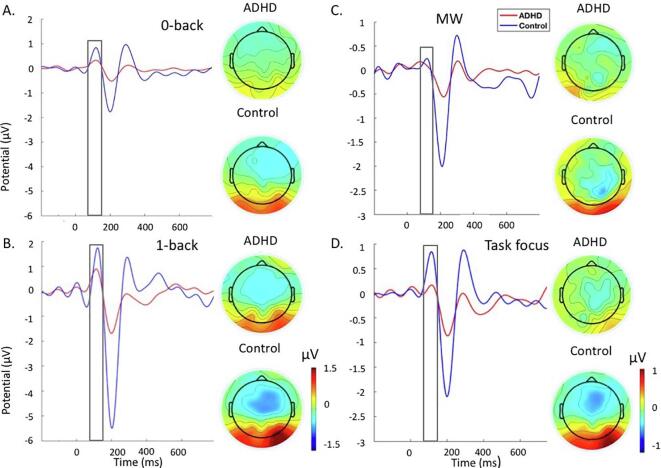
Grand average stimulus-locked event-related potentials of the P1 at the parieto-occipital electrodes at 80 to 150 ms in the ADHD group (red) and control group (blue) across the 1 back and 0-back conditions during the MWT. A. Grand average and topographic maps for the 0-back. B. Grand average and topographic maps for the 1-back. C. Grand average and topographic maps for the MW episodes. D. Grand average and topographic maps for the task focus. Abbreviations: ADHD-attention-deficit/hyperactivity disorder, MW-mind wandering. (For interpretation of the references to colour in this figure legend, the reader is referred to the web version of this article.)

**Fig. 3:**
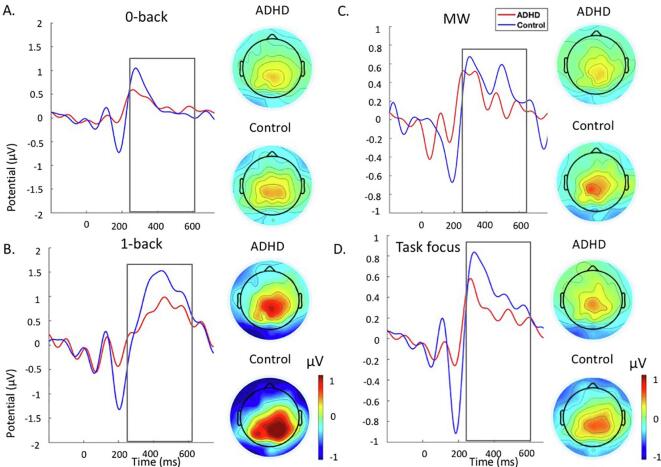
Grand average stimulus-locked event-related potentials of the P3 at the centro-parietal electrodes at 250 to 650 ms ADHD group (red) and control group (blue) across the 1 back and 0-back conditions during the MWT. A. Grand average and topographic maps for the 0-back. B. Grand average and topographic maps for the 1-back. C. Grand average and topographic maps for the MW episodes. D. Grand average and topographic maps for the task focus. Abbreviations: ADHD-attention-deficit/hyperactivity disorder, MW-mind wandering. (For interpretation of the references to colour in this figure legend, the reader is referred to the web version of this article.)

**Fig. 4:**
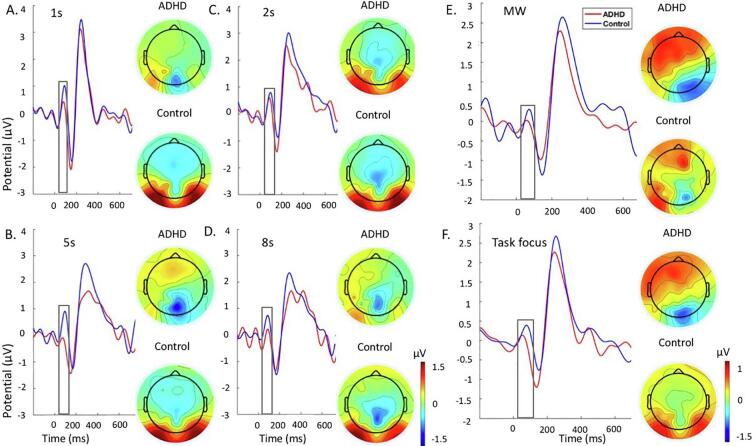
Grand average stimulus-locked event-related potentials of the P1 at the parieto-occipital electrodes at 80 to 130 ms ADHD group (red) and control group (blue) across the 1 s, 2 s, 5 s and 8 s delays during the SAT. A. Grand average and topographic maps for the 1 s. B. Grand average and topographic maps for the 5 s. C. Grand average and topographic maps for the 2 s. D. Grand average and topographic maps for the 8 s. E. Grand average and topographic maps for the MW episodes. F. Grand average and topographic maps for the task focus. Abbreviations: ADHD-attention-deficit/hyperactivity disorder, MW-mind wandering. (For interpretation of the references to colour in this figure legend, the reader is referred to the web version of this article.)

**Fig. 5:**
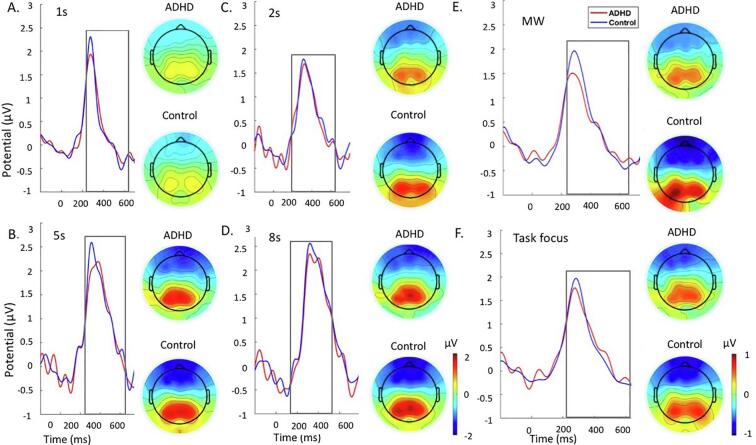
Grand average stimulus-locked event-related potentials of the P3 at the centro-parietal electrodes at 250 to 650 ms ADHD group (red) and control group (blue) across the 1 s, 2 s, 5 s and 8 s delays during the SAT. A. Grand average and topographic maps for the 1 s. B. Grand average and topographic maps for the 5 s. C. Grand average and topographic maps for the 2 s. D. Grand average and topographic maps for the 8 s. E. Grand average and topographic maps for the MW episodes. F. Grand average and topographic maps for the task focus. Abbreviations: ADHD-attention-deficit/hyperactivity disorder, MW-mind wandering. (For interpretation of the references to colour in this figure legend, the reader is referred to the web version of this article.)

For both analyses, the data were segmented in epochs around the stimulus between −1000 and 1000 ms using the −200-ms pre-stimulus period for baseline correction. P1 and P3 were identified within the selected electrodes and latency windows for which effects were expected to be largest, based on previous ADHD and MW studies using attentional and working memory tasks (Michelini et al., 2018, James et al., 2019, Bozhilova et al., 2020a, Kim et al., 2014, Gomarus et al., 2009, Smallwood et al., 2009, Smallwood et al., 2012, Baird et al., 2014, Wiersema et al., 2006). These parameters were also verified against the topographic maps and the grand averages (Fig. 2, Fig. 3, Fig. 4, Fig. 5). ERPs were quantified as mean amplitudes within selected windows, which eliminates the effect of peak latency variability (Luck, 2005). Following previous similar studies, P1 was measured over parieto-occipital regions (average of electrodes: PO7, PO3, PO4, PO8) between 80 and 150 ms (Nazari et al., 2010, Gonen-Yaacovi et al., 2016) in the MWT and between 80 and 130 ms (Dockree et al., 2017) in the SAT, given latency differences in P1 between tasks (Fig. 2, Fig. 4). P3 was measured at 250 to 650 ms over centro-parietal regions (average of electrodes: CP1, CP2, CPz, P1, P2, Pz) in both tasks (Fig. 3, Fig. 5).

Only participants with at least 20 artefact-free EEG segments in each condition or probe were included in the ERP analyses, since at least 20 artefact-free EEG segments are required to observe reliable neural effects and obtain valid ERP indices (Rietdijk et al., 2014). For Analysis 1, from the original sample of 56 participants, four individuals with ADHD were excluded because of extremely large movement artefacts. Four controls were also excluded due to corrupted data files or poor data quality. Analysis 1 included 23 individuals with ADHD and 25 controls. For Analysis 2*,* additional participants were excluded due to not having sufficient trials for analyses contrasting MW and task focus periods. For the MWT, 4 additional controls had no MW episodes and 2 individuals with ADHD had no task focus episodes, resulting in 21 individuals with ADHD and 21 controls. For the SAT, 7 controls did not have enough MW episodes, resulting in 23 individuals with ADHD and 18 controls.

### 2.5 Statistical analyses

To study ERP components during task conditions (Analysis 1), we tested the effects of condition (1-back/0-back for MWT; 1 s/2s/5s/8s for SAT), group (ADHD/controls) and condition-by-group interactions on each ERP in repeated measures general linear models. To test the effect of MW frequency on the ERP variables, we repeated these analyses adding MW frequency as a covariate. Controlling for the effect of MW in the model allowed us to directly assess whether group differences in ERPs and task performance could be attributed to the diagnosis of ADHD, or to the higher occurrence of MW frequency in the ADHD group. To understand the effect of MW and task focus on ERP components and task performance (Analysis 2), we tested the effects of probe (MW/task focus), group (ADHD/Controls) and probe-by-group interactions on each ERP and performance measures in repeated measures general linear models. In both analyses, we ran post-hoc tests comparing groups in each condition/probe separately and comparing conditions in each group even in the absence of significant condition-by-group or probe-by-group interactions, which our sample may be underpowered to detect. In an additional analysis, due to a potential temporal relationship between P1 and P3, we entered P1 as a covariate in Analyses 1 and 2.

In the MWT, we ran analyses across target and non-target trials, as there were large, positive correlations between the two stimulus types (Supplementary Analysis 1, Supplementary Table S3) and we expected that condition and MW would impact the ERPs in targets and non-target trials to a similar extent. Supplementary Analysis 2 and Supplementary Table S4 report analyses run in target and non-target trials for completeness, showing comparable results for both stimulus types.

All ERP measures were normally distributed. Cohen’s d with correction for small sample sizes (n < 50) was computed for between-group and within-group comparisons (Lakens, 2013). We used a false discovery rate (FDR) threshold for the between and the within-group effects in Analysis 1 and 2 to account for multiple testing (Table 2). FDR significant p-values were p ≤ 0.026 for Analysis 1 and p ≤ 0.018 for Analysis 2. The within- and the between-group effects not surviving FDR correction and with p < 0.05 are presented as trend-level effects that require further research. Results are interpreted based on both p-values and Cohen’s d. All analyses were conducted in SPSS 24 (IBM Corporation, Somers, NY).

**Table 2:** Main and interaction effects from general linear repeated measures models.

	*MWT*						*SAT*					
Analysis 1	Group		Condition		Group × condition		Group		Delay		Group × delay	
	F	p	F	p	F	p	F	p	F	p	F	p
P1	5.73	0.021*	12.92	0.001*	0.06	0.805	9.18	0.005*	7.67	0.001*	3.46	0.019*
P3	5.39	0.025*	42.57	0.001*	4.53	0.039*	2.03	0.245	80.25	0.001*	2.40	0.079

Analysis 2	Group		Probe		Group × probe		Group		Probe		Group × probe	
	F	p	F	p	F	p	F	p	F	p	F	p

P1	4.24	0.002*	1.04	0.464	2.53	0.314	0.78	0.607	1.08	0.167	4.10	0.047*
P3	5.16	0.027*	0.96	0.475	0.15	0.882	0.62	0.314	0.04	0.278	0.39	0.082
MRT	6.83	0.012*	10.65	0.002*	0.01	0.992	8.99	0.005*	56.04	0.001*	0.01	0.934
RTV	3.04	0.088	4.13	0.049*	4.22	0.046*	5.43	0.026*	57.55	0.001*	0.35	0.555
Errors	0.36	0.549	31.41	0.027*	14.68	0.002*	8.31	0.007*	10.15	0.003*	15.12	0.001*

Abbreviations: MWT-Mind Wandering task, SAT-Sustained Attention Task, MRT-Mean Reaction Time, RTV-Reaction Time Variability.

Notes: *p < 0.05. General linear repeated measures models tested for main effects of group (ADHD vs controls), condition (in the MWT, 1-back vs 0-back), delay (in the SAT, 1 s, 2 s, 5 s, 8 s) or probe (MW vs task focus), and two-way interactions (group-by-condition, group-by-delay or group-by-probe) on ERP (P1, P3) and task performance (RTV, MRT, Errors) measures.

Given the novelty of the current study, formal a-priori power calculations indicate 80% power to detect medium effects sizes (d > 0.70) as statistically significant (α = 0.02) with the current sample (n = 48). The calculation refers to the statistical models used with and without covariates using the statistical software, G*Power (Faul et al., 2007).

## 3 Results

All main and interaction effects are reported in Table 2. Here, we focus on both FDR-significant and trend-level between- and within-group post-hoc comparisons.

### 3.1 Analysis 1: low versus high demand

#### 3.1.1 P1

P1 during the MWT (Fig. 2) was attenuated in individuals with ADHD compared to controls in the 0-back at trend level (not surviving FDR corrections), but did not differ between groups in the 1-back condition (Table 3). Both groups showed significantly larger P1 in the 1-back compared to the 0-back condition (Table 3). When we covaried for MW in the analysis, the between-group difference on P1 during 0-back was no longer a trend, although the effect size was slightly reduced (Table 2). The within-group differences between conditions remained significant in both groups after adding MW as a covariate (Table 3).

**Table 3:** Comparisons between and within groups on ERP measures during task conditions.

		Between-group comparisons							
		ADHD vs Control				ADHD vs Control (covarying MW)			
		*d*		p		*d*		p	
*MWT*									
P1	*1back*	0.45		0.070		0.40		0.368	
	*0back*	*0.60*		0.041‡		0.45		0.262	
P3	*1back*	*0.60*		0.026*		0.47		0.157	
	*0back*	0.28		0.118		0.28		0.951	
*SAT*									
P1	*1s*	**1.06**		0.003*		*0.76*		0.100	
	*2s*	0.33		0.321		0.17		0.798	
	*5s*	**1.27**		0.001*		**0.83**		0.053	
	*8s*	**0.91**		0.010*		*0.51*		0.143	
P3	*1s*	0.14		0.640		0.08		0.632	
	*2s*	0.44		0.130		0.40		0.334	
	*5s*	0.43		0.195		0.34		0.628	
	*8s*	0.37		0.217		0.26		0.781	

Within-group comparisons									

		ADHD		ADHD (covarying MW)		Controls		Controls (covarying MW)	
		*d*	p	*d*	p	*d*	p	*d*	p

*MWT*									
P1	*1back* vs *0back*	*0.57*	0.016*	*0.63*	0.009*	*0.51*	0.018*	0.48	0.027*
P3	*1back* vs *0back*	*0.73*	0.002*	*0.79*	0.001*	***1.12***	0.001*	***1.04***	0.001*
*SAT*									
P1	*2s* vs *5s*	**0.99**	0.001*	**0.85**	0.001*	0.08	0.709	0.11	0.425
	*2s* vs *8s*	0.36	0.103	0.31	0.159	0.02	0.943	0.15	0.604
	*5s* vs *8s*	0.47	0.035‡	0.47	0.038‡	0.03	0.875	0.15	0.488
P3	*2s* vs *5s*	**0.88**	0.001*	*0.63*	0.012*	**0.92**	0.001*	**0.81**	0.001*
	*2s* vs *8s*	**1.32**	0.001*	**1.11**	0.001*	**1.46**	0.001*	**1.21**	0.001*
	*5s* vs *8s*	*0.58*	0.019*	**0.84**	0.003*	*0.64*	0.004*	*0.53*	0.018*

Abbreviations: MWT-Mind Wandering task, SAT-Sustained Attention Task, MW-Mind Wandering.

Notes: *FDR correction significant at p ≤ 0.026, ‡trend-level effects at p < 0.05. **Bold**: d ≥ 0.80 indicating large effect size, *Italics*: d ≥ 0.50 indicating a medium effect size.

During the SAT, individuals with ADHD had a significantly lower P1 amplitude in the 1 s, 5 s and 8 s delays compared to controls (i.e., surviving FDR corrections), but there were no group differences in the 2 s (Fig. 4; Table 3). Controls did not show differences in P1 between the longer delays (Table 3). In contrast, individuals with ADHD showed smaller P1 in the 5 s compared to 2 s and 8 s, although the difference between 5 s and 8 s did not survive FDR correction (Table 3). After adding MW as a covariate, statistical between-group differences for 1 s, 5 s and 8 s were no longer significant. The effect sizes were also reduced from large to medium in the 1 s and 8 s delay, but they remained large in the 5 s delays (Table 3). The within-group differences remained significant when covarying for MW.

#### 3.1.2 P3

Individuals with ADHD had significantly smaller P3 in the 1-back condition compared to controls during the MWT (i.e., surviving FDR correction), but there were no differences in the 0-back condition (Fig. 3; Table 3). Both groups had significantly larger P3 in the 1-back compared to the 0-back condition (Table 3) (i.e., surviving FDR correction). After adding MW frequency as a covariate, the statistical difference between groups was no longer significant, although the effect size was only slightly reduced. The within-group differences remained significant after adding MW frequency as a covariate (Table 3). Adding P1 as a covariate did not alter the results (Supplementary Analysis 3 and Supplementary Table S5).

There were no group differences in P3 in any delay condition during the SAT (Fig. 5, Table 3). Both groups showed significantly increasing P3 with increasing delays (i.e., surviving FDR correction) (Table 3). When we added MW and P1 separately as covariates, the results remained unchanged (Table 2, Supplementary Analysis 3 and Supplementary Table S5).

### 3.2 Analysis 2: MW versus task focus

#### 3.2.1 P1

Both during the MWT and the SAT, individuals with ADHD had significantly smaller P1 compared to controls, which also survived FDR correction, during task focus, but not during MW episodes (Table 4). Controls showed lower P1 during MW compared to task focus, whereas individuals with ADHD did not show differences across conditions (Table 4). However, this within-group effect for P1 between MW and task focus in controls did not survive FDR correction in the MWT and warrants replication.

**Table 4:** Comparisons between and within groups for all ERP measures and task performance during periods of MW and task focus.

	Between-group comparisons				
		ADHD vs Controls			
		*d*		p	
*MWT*					
P1	Task focus	**1.05**		0.001*	
	MW	0.48		0.137	
P3	Task focus	*0.65*		0.025‡	
	MW	*0.56*		0.034‡	
MRT	Task focus	**0.91**		0.002*	
	MW	*0.68*		0.026‡	
RTV	Task focus	0.21		0.457	
	MW	*0.72*		0.018*	
Errors	Task focus	0.30		0.297	
	MW	**1.11**		0.001*	
*SAT*					
P1	Task focus	**0.86**		0.007*	
	MW	0.05		0.810	
P3	Task focus	0.04		0.850	
	MW	**0.92**		0.011*	
MRT	Task focus	**2.23**		0.001*	
	MW	*0.51*		0.119	
RTV	Task focus	**1.62**		0.001*	
	MW	0.32		0.322	
Errors	Task focus	*0.58*		0.056	
	MW	**1.15**		0.001*	

	Within-group comparisons				
		ADHD		Controls	
		d	p	d	p

*MWT*					
P1	Task focus vs MW	0.08	0.711	*0.49*	0.033‡
P3	Task focus vs MW	0.08	0.683	0.22	0.337
MRT	Task focus vs MW	*0.61*	0.006*	0.39	0.075
RTV	Task focus vs MW	*0.73*	0.002*	0.01	0.989
Error	Task focus vs MW	*0.63*	0.005*	0.38	0.085
*SAT*					
P1	Task focus vs MW	0.16	0.439	**0.98**	0.014*
P3	Task focus vs MW	0.08	0.694	0.11	0.667
MRT	Task focus vs MW	**1.17**	0.001*	**1.17**	0.001*
RTV	Task focus vs MW	**1.17**	0.001*	**1.19**	0.001*
Errors	Task focus vs MW	**0.88**	0.001*	0.25	0.302

Abbreviations: MWT-Mind Wandering task, SAT-Sustained Attention Task, MW-Mind Wandering Episodes, MRT-Mean Reaction Time, RTV-Reaction Time Variability.

Notes: *FDR correction significant at p ≤ 0.018, ‡trend-level effects at p < 0.05. **Bold**: d ≥ 0.80 indicating large effect size, *Italics*: d ≥ 0.50 indicating a medium effect size. Analyses 2 included 21 controls and 21 individuals with ADHD in the MWT, and 18 controls and 23 individuals with ADHD in the SAT.

#### 3.2.2 P3

Individuals with ADHD showed smaller P3 compared to controls, although at trend level (i.e., not surviving FDR correction), in the MWT during both MW and task focus (Fig. 3, Table 4). Neither group showed differences across conditions (Table 3). The group difference during MW, but not during task focus, was no longer significant after adding P1 as a covariate, although the effect size was only slightly reduced (Supplementary Analysis 3 and Supplementary Table S6).

During the SAT, individuals with ADHD had significantly smaller P3 than controls during MW episodes, but not during task focus (Table 4) (i.e., surviving FDR correction). Neither group showed differences across conditions (Table 4). The group differences were no longer significant after adding P1 as a covariate, and the effect size during MW was reduced from large to small (Supplementary Analysis 3 and Supplementary Table S6).

#### 3.2.3 Task performance

During the MWT, individuals with ADHD showed higher MRT during periods of both MW and task focus compared to controls, although this effect for MW periods did not survive FRD correction (Table 4). RTV was significantly higher in the ADHD compared to the control group during MW, but not during task focus in the MWT (Table 4). During the SAT, individuals with ADHD showed significantly higher MRT and RTV than control during task focus, but not during MW. Across tasks, compared to controls, individuals with ADHD made significantly more errors than controls during MW, but not during task focus. All between-group effects for RTV and error rate survived FDR corrections. The ADHD group showed significantly worse performance (i.e., higher MRT, RTV and error rate) during MW compared to task focus across tasks, which survived FDR corrections, while the control group showed significantly higher MRT and RTV during MW compared to task focus during the SAT, but not during the MWT (Table 3).

## 4 Discussion

This is the first study to investigate modulations of neural activity of early sensory and attentional processes in relation to MW frequency, during varying task conditions, and periods of MW and task focus, in individuals with and without ADHD. Compared to controls, adults with ADHD showed attenuations in P1 during high demands on sustained attention, and attenuation in P3 during high demands on working memory. These group differences were explained by MW frequency, which was higher in adults with ADHD compared to controls. Individuals with ADHD also showed reduced P1 relative to controls during task focus, but not during MW episodes across tasks, and reduced P3 during MW, but not during task focus in the SAT. These findings show that differences between groups on modulations of early sensory processes are linked to modulations of MW frequency, whereas modulations in attention allocation appear task- and condition-dependent. Taken together, this study provides converging evidence that higher MW frequency and inefficient adjustment from MW episodes to task focus may contribute to deficient neural activity of sensory processing in individuals with ADHD compared to controls.

Our first analysis sought to understand the relationship between MW frequency and sensory processing (P1) and attention allocation (P3) during varying task conditions in individuals with ADHD and controls (Analysis 1). Results for P1 were largely consistent with our hypotheses. Across tasks, adults with ADHD compared to controls showed attenuated P1, although this effect survived multiple testing corrections only in the SAT, during conditions that we previously found associated with the highest level of MW frequency in the ADHD group (0-back of the MWT; 5 s and 8 s delays of the SAT) (Bozhilova et al., 2020b. The lack of difference from controls during the 2 s delays is also consistent with our previous behavioural findings in this sample, showing less widespread differences in task performance between ADHD and control groups during the 2 s delays than during the longer (5 s, 8 s) delays. Individuals with ADHD compared to controls also showed impaired sensorimotor function, as indicated by reduced P1 during the 1 s, placing the highest demand on sensorimotor function (Christakou et al., 2013). Regarding attention allocation (P3), in contrast with our predictions, adults with ADHD showed decreased P3 during the 1-back condition, which we previously found associated with lower MW frequency (Bozhilova et al., 2020b). In contrast, no group differences emerged for P3 across all delays in the SAT. Our P3 findings are in line with previous studies on adults with ADHD showing reduced P3s during challenging conditions posing high demands on executive functions during working memory, attentional and inhibitory tasks (Kim et al., 2014, Bozhilova et al., 2020a, Michelini et al., 2016, Michelini et al., 2018), but not during less challenging conditions. P3 attenuations in ADHD might therefore only emerge in conditions requiring higher engagement of executive functions. Of note, the statistical differences between groups were no longer significant when accounting for differences in MW frequency in the analyses of P1 and P3. However, the effect sizes remained medium to large, suggesting that increased MW frequency might only partly explain the neural deficits underlying sensory and attentional processes in individuals with ADHD. This might be especially the case under high demand on working memory since the effect sizes in the MWT were only slightly reduced. Conversely, effect sizes were reduced more substantially when controlling for MW in the SAT, especially following 8 s delays, suggesting that increased MW frequency in individuals with ADHD might have the most detrimental effect on neural processes under very high demands on sustained attention.

We also examined modulations of P1 and P3 within each group with changing task demands. In adults with and without ADHD, we found that neural activity (P1, P3) was higher during 1-back compared to the 0-back condition (i.e., context regulation of neural activity), consistent with previous findings on P1 (Kim et al., 2014, Geden et al., 2018). This finding parallels our previous behavioural findings showing context regulation of MW frequency (i.e., lower MW frequency during the 1-back compared to the 0-back) in adults with ADHD, and continuous task focus across conditions (0-back and 1-back) in controls (Bozhilova et al., 2020b). The finding that controls showed, similar to the ADHD group, a difference in P1 and P3 between task conditions supports our previous propositions that context regulation of neural activity would be present in the control group even in the absence of behavioural modulation of MW with changing task demands (Bozhilova et al., 2020b). In the SAT, controls showed comparable sensory processing (P1) across delays (2 s, 5 s, 8 s), while individuals with ADHD showed poorer sensory processing during the 5 s compared to the 2 s and 8 s delays, although the differences between 5 s and 8 s did not reach statistical significance after correcting for multiple testing. Future studies with larger sample sizes therefore need to replicate this effect. Both groups showed increasing attention allocation (P3) with increasing delays (2 s, 5 s, 8 s in order of increase), suggesting that P3 is modulated by the level of cognitive demand specific for each task condition. The findings for P1, but not P3, parallel our previous behavioural findings in the SAT, as controls showed continuous task focus with increasing delays (2 s, 5 s, 8 s), reflecting context regulation of both MW and neural activity (Bozhilova et al., 2020b). Instead, adults with ADHD showed increasing MW frequency with increasing delays (2 s, 5 s, 8 s), reflecting deficient context regulation (Bozhilova et al., 2020b). This finding in individuals with ADHD parallels a well-established lack of neural adaptation with cognitive demands in individuals with ADHD (Christakou et al., 2013, Bollmann et al., 2017, Michelini et al., 2019, Vatansever et al., 2019). MW frequency, however, did not explain any of these modulations of P1 and P3 with changing task demands in either group. This finding suggests that modulations of neural activity in each group are modulated by the cognitive demand evoked by each task.

Our second analysis further assessed the relationship of MW to neural activity (P1, P3) more directly by contrasting periods of MW and task focus (Analysis 2). During both tasks, adults with and without ADHD showed comparably low P1 during MW, indicating perceptual decoupling. This result is in line with the findings of Analysis 1, showing that differences between ADHD and control groups on P1 are explained with MW frequency. Importantly, compared to controls, individuals with ADHD showed lower P1 than controls during task focus, suggesting that they remained in a state of perceptual decoupling during task focus. This key group difference suggests that poorer adaptation of sensory processing might be a primary deficit in individuals with ADHD. Individuals with ADHD further showed lower attention allocation (P3) than controls during both MW and task focus in the MWT, although at trend level. This trend-level pattern supports our interpretation of the findings contrasting task conditions (Analysis 1) that the P3 is modulated by the cognitive demand evoked by the task condition and is unrelated to context regulation of MW. This finding suggests that individuals with ADHD, unlike controls, might have difficulty allocating additional attention during high working memory demands (1-back condition), which require continuous attention allocation to both non-targets (less salient) and targets (more salient). During the SAT, individuals with ADHD compared to controls had reduced attention allocation during MW, but not during task focus. These findings suggest that P3 reductions are malleable rather than fixed in individuals with ADHD and suggest that improvements might be possible with task manipulations that reduce MW frequency. In line with this pattern, it has been shown that P3 attenuations improve in individuals with ADHD under faster and rewarded conditions (Cheung et al., 2017). After controlling for P1, however, all group differences on P3 in both tasks were no longer significant, suggesting that perceptual decoupling might affect subsequent attention allocation during periods of MW. Since the effect sizes were only slightly reduced in the MWT, but more substantially reduced from large to small in the SAT, P1 might have a greater impact on P3 during tasks placing high demand on sustained attention.

Within-group comparisons further showed an improvement in P1 from MW to task focus in controls across tasks, although at trend level in the MWT. Instead, individuals with ADHD did not show a difference in P1 between MW and task focus, indicating deficient context regulation of sensory processing. Together with the differences in P1 between groups, these findings in the ADHD group suggest a lack of neural adaption from conditions of low alertness, such as MW (Braboszcz and Delorme, 2011, Smallwood and Schooler, 2006), to task focus, in line with evidence of deficient neural adaptation from rest to task in ADHD (Skirrow et al., 2015, Rommel et al., 2016). With regard to the P3, both groups showed no change in P3 from MW to task focus across tasks, further supporting a lack of association between P3 and context regulation of MW.

With regard to task performance, our findings indicate that individuals with ADHD in the MWT showed comparable response variability (RTV) and error rate to controls during periods of task focus, but significantly worse performance during MW. This pattern in task performance appears parallel to context regulation of both neural activity (P1, P3) and MW frequency in both groups during the MWT. In contrast, compared to controls, individuals with ADHD had increased MRT during both task focus and MW during the MWT, although the latter effect was at trend level and requires replication. Compared to controls, individuals with ADHD also made more errors during MW, but not during task focus across tasks. This pattern further supports the detrimental impact of increased MW frequency on task accuracy in individuals with ADHD. During the SAT, individuals with ADHD performed worse (increased MRT, RTV) than controls during task focus, but not during MW. Both groups also showed similar reaction times during MW compared to task focus in the SAT, but not in the MWT, indicating that MW periods might be particularly impairing to performance during this task placing increasing demands on sustained attention. This finding supports the notion that MW reduces attentional resources at the cost of task performance (Smallwood, 2010) and confirms with previous findings in community samples (Forster and Lavie, 2009, Stawarczyk et al., 2011, Smallwood et al., 2013, Smallwood et al., 2008, Kam and Handy, 2014). Adults with ADHD, but not controls, further showed lower accuracy during MW periods than during task focus in both tasks, supporting the finding that during MW episodes individuals with ADHD made more errors compared to controls across tasks.

## 5 Implications

This study aimed to investigate the neural and cognitive mechanisms through which MW might play a role in ADHD symptoms and impairments. More specifically, the absence of improvement in basic, pre-attentive, perceptual processes (e.g., early visual processing) from episodes of MW to task focus might facilitate inattentive behaviours (e.g., careless mistakes, sustaining attention on everyday tasks or failure to follow on through with tasks/activities) interfering with daily function in adults with ADHD. Future work could therefore evaluate the impact of deficient early sensory processing and increased MW frequency on both experimental and everyday performance and suggest treatment strategies which target early perceptual processing in ADHD. For example, the use of stimulant medication has been linked to normalisation of neural activity from rest to task (Rubia et al., 2014, Skirrow et al., 2015), suggesting that context regulation of MW and associated neural activity (e.g., P1) might serve as potential biomarker of treatment response. Due to the strong association between visual attention, MW and clinical outcomes (Lenartowicz et al., 2018, Mowlem et al., 2016), experience sampling measures of MW and associated neural markers of visual attention may be useful targets for real-time monitoring of the treatment response.

## 6 Limitations and future directions

One limitation of this study is that controls had significantly less MW episodes compared to the ADHD group, and a large proportion of controls had no MW episodes (Bozhilova et al., 2020b). In addition, the sample size is small and could only detect medium-to-large effects (*d* > 0.50 to d > 0.80). This likely explains why several interaction effects were non-significant and some post-hoc effects did not survive multiple testing corrections. Although MW frequency as a covariate might have driven some of the effects, the study may have been underpowered to detect small independent effects of MW and ADHD status. While our analysis did not directly test the mechanistic hypothesis, our findings provide convergent evidence for the association of MW with P1 in individuals with ADHD, and highlight the need for future research using causal modelling. Future research should confirm these findings in a larger sample and using tasks that generate greater variability in MW episodes, in order to detect subtle effects that could not be detected with the current study design. In addition, the MWT was relatively easy and future studies could examine the effect of higher demand on working memory (i.e., 2/3-back) and lower demand on sustained attention (i.e., long, same-length, predictable intervals) on the frequency of MW in individuals with and without ADHD. Another limitation is the lower number of artefact-free trials across conditions in individuals with ADHD compared to controls. Individuals with ADHD had a significantly lower number of trials across all conditions, but between and within-group effects emerged only for P3 in the 1-back, and for P1 in the 1 s, 5 s and 8 s, suggesting that the number of trials might not have impacted our results (Supplementary Table S2). Future work should aim to equate the number of trials across groups to avoid signal to noise ratio (SNR) effects, for example by using tasks with a greater number of trials. Future work may also benefit from using measures sensitive to the changes associated with the onset of MW and its progression during the task, such as pupil diameter (Pelagatti et al., 2020), without affecting the natural flow of MW with experience-sampling probes. Nevertheless, the inclusion of probes is an unlikely explanation for the deficient context regulation of MW and associated neural activity during the SAT in the ADHD group. In particular, the number and frequency of probes was identical across tasks, but individuals with ADHD did not report identical MW frequency across tasks. Further, MW frequency was found to be comparable across self-report, experimental and daily-life experience-sampling measures in both clinical (Moukhtarian et al., 2020) and non-clinical populations (McVay et al., 2009), suggesting that the inclusion of probes did not drive the effects.

## 7 Conclusions

This study found that adults with and without ADHD showed low P1 during periods of MW, but the ADHD group showed lower P1 than controls during task focus, suggesting that poor adaptation of sensory processing might be a primary deficit in ADHD. The modulations in early sensory processing (P1) in response to task demands appear to parallel modulations in MW frequency (Bozhilova et al., 2020b). However, both sensory (P1) and attention (P3) processing appear to be primarily responses to the level of cognitive demand evoked by the task conditions. The study findings also provide convergent evidence that P1 reductions in individuals with ADHD compared to controls may be explained by increased MW frequency and reflect inefficient adjustment in early sensory responses from periods of MW to periods of task focus. Since pharmacological treatment (Skirrow et al., 2015, Rubia et al., 2014) and high-salience conditions (Liddle et al., 2011, Cheung et al., 2017) have been shown to ‘normalise’ neural adaptation to task demands and from rest to task, future studies may examine whether these factors have a similar effect on adaptations of pre-attentive sensory processing related to MW.

## Declaration of Competing Interest

The authors declare the following financial interests/personal relationships which may be considered as potential competing interests: Professor Jonna Kuntsi has given talks at educational events sponsored by Medice: all funds are received by King’s College London and used for studies of ADHD. Professor Philip Asherson has received honoraria for consultancy to Shire/Takeda, Flynn-Pharma, Eli-Lilly, Janssen, Novartis, Lundbeck and Medice; educational/research awards from Janssen, Shire, Lilly, Novartis, Flynn Pharma, Vifor Pharma, GW Pharma and QbTech; speaker at sponsored events for Shire/Takeda, Lilly, Novartis, Medice, Janssen-Cilag and Flynn Pharma.

Professor Katya Rubia has received a grant from Shire/Takeda for another project.
