# Supplementary material for: Electrophysiological modulation of sensory and attentional processes during mind wandering in attention-deficit/hyperactivity disorder
Source: Neuroimage Clin. 2020 Dec 30;29:102547. doi: 10.1016/j.nicl.2020.102547 (PMC7808945; doi:10.1016/j.nicl.2020.102547)
Supplement: Supplementary data 1 [file mmc1.docx]

**SUPPLEMENTARY MATERIALS**

**Supplementary analyses**

In the working memory condition (1-back) of the Mind Wandering Task (MWT), ERPs to non-targets and targets represent two aspects of working memory (encoding and retrieval respectively). Previously, electrophysiological correlates (e.g., occipital alpha activity and P3) underlying attentional processes were found to underlie these two aspects of working memory (Kim, Liu, Glizer, Tannock, & Woltering, 2014).

To test whether the two stimulus types reflect the same working memory aspects, we performed Pearson’s correlations between each stimulus type in each condition. Significant correlations would suggest that the two stimulus types reflect similar processes (Supplementary Analysis 1).

In additional analyses, we first examined the effect of condition (1-back/0-back), group (ADHD/Control) and condition-by-group interaction on all ERP measures (P1, P3) from target and non-target trials separately using repeated-measures general linear models (Supplementary Analysis 2). Comparable results for target and non-targets would suggest the two stimulus types reflect the same processes.

Due to a potential temporal relationship between P1 and P3, we repeated Analyses 1 and 2 in the main text controlling for the effect of P1 on P3 (Supplementary Analysis 3 and Supplementary Table 6).

**Supplementary results**

**Supplementary Analysis 1**

Pearson’s correlations between stimulus types showed that there was a strong and positive correlation between all ERP measures (Supplementary Table 3).

**Supplementary Analysis 2**

**P1**

There was a main effect of group (p=0.011), but there was no main effect of condition (p=0.804) or condition-by-group interaction (p=0.076) on P1 in target trials. Individuals with ADHD had a smaller P1 amplitude compared to controls.

There was a main effect of group (p=0.006), but there was no main effect of condition (p=0.500) or condition-by-group interaction (p=0.934) on P1 in non-target trials. Individuals with ADHD had a smaller P1 amplitude compared to controls.

**P3**

There was a main effect of condition (p=0.001) and group (p=0.047), but there was no significant condition-by-group interaction (p=0.321) on the P3 in target trials. P3 was attenuated during the 0-back compared to the 1-back condition. Individuals with ADHD had a smaller P3 compared to controls.

There was a main effect of condition (p=0.001) and group (p=0.048), but there was a borderline significant condition-by-group interaction (p=0.051) on the P3 in non-target trials. P3 was attenuated during the 0-back compared to the 1-back condition. Individuals with ADHD had a smaller P3 compared to controls.

Post-hoc analyses showed a differential pattern across conditions (Supplementary Table 4), but they are likely to reflect insufficient power to detect medium and small effects.

**Supplementary Analysis 3**

After adding P1 as covariate in the analysis of P3, group differences in P3 were no longer significant in the context of MW and task focus during both tasks (Supplementary Table 5). No other changes in results were detected.

**Supplementary Table 1.** Group comparisons for behavioural measures

|  | | | **ADHD** | | **Control** | | ***d*** | **p** | | |
| --- | --- | --- | --- | --- | --- | --- | --- | --- | --- | --- |
|  | |  | | **Mean ± SD** | **Mean ± SD** |  | | |  |  |
| ***MWT*** | | | | | | | | | |  |
| MW Frequency | 1-back | | | 0.45±0.30 | 0.10±0.12 | **1.52** | | | <0.001*** |  |
|  | 0-back | | | 0.66±.0.21 | 0.16±0.19 | **2.45** | | | <0.001*** |  |
| MRT | 1-back | | | 1204.18±292.25 | 938.71±233.27 | **0.99** | | | 0.001*** |  |
|  | 0-back | | | 1105.05±356.92 | 824.64±215.73 | **0.95** | | | 0.001*** |  |
| RTV | 1-back | | | 544.41±141.04 | 341.67±139.83 | **1.41** | | | <0.001*** |  |
|  | 0-back | | | 468.38±254.55 | 288.33±172.56 | **0.83** | | | 0.004* |  |
| Errors | 1-back | | | 0.59±0.37 | 0.40±0.35 | *0.52* | | | 0.090 |  |
|  | 0-back | | | 0.30±0.27 | 0.36±0.32 | 0.20 | | | 0.480 |  |
| ***SAT*** | | | | | | | | | |  |
| MW Frequency | 2s | | | 0.53±0.32 | 0.13±0.19 | **1.52** | | | <0.001*** |  |
|  | 5s | | | 0.69±0.31 | 0.13±0.17 | **2.24** | | | <0.001*** |  |
|  | 8s | | | 0.68±0.34 | 0.18±0.19 | **1.83** | | | <0.001*** |  |
| MRT | 1s | | | 315.14±25.30 | 287.45±31.33 | **0.93** | | | 0.001*** |  |
|  | 2s | | | 379.00±29.18 | 370.48±37.76 | 0.23 | | | 0.381 |  |
|  | 5s | | | 395.70±25.71 | 378.01±33.30 | *0.55* | | | 0.050 |  |
|  | 8s | | | 406.23±27.69 | 379.49±34.29 | **0.81** | | | 0.003** |  |
| RTV | 1s | | | 67.61±9.84 | 55.45±9.10 | **1.24** | | | <0.001*** |  |
|  | 2s | | | 52.69±11.31 | 49.25±11.66 | 0.30 | | | 0.291 |  |
|  | 5s | | | 51.70±8.27 | 50.54±10.33 | 0.11 | | | 0.660 |  |
|  | 8s | | | 50.37±10.93 | 51.20±8.12 | 0.10 | | | 0.762 |  |
| Errors | 1s | | | 0.05±0.04 | 0.02±0.02 | **0.95** | | | <0.001*** |  |
|  | 2s | | | 0.06±0.04 | 0.04±0.03 | *0.56* | | | 0.050 |  |
|  | 5s | | | 0.08±0.05 | 0.05±0.04 | *0.65* | | | 0.010** |  |
|  | 8s | | | 0.09±0.04 | 0.06±0.04 | *0.73* | | | 0.020** |  |

Abbreviations: ADHD- Attention-deficit/hyperactivity disorder, MRT – mean reaction time, RTV- reaction time variability.

Notes: * significant at p≤0.032, **significant at p≤.05, ***significant at p≤.001, **Bold**: d≥.80 indicating large effect size, Italics: d≥.50 indicating a medium effect size, d≥.20 indicating a small effect size. Errors during the SAT have been calculated by dividing the total number of omission errors by the number of trials. These results have been reported in a previous publication (Bozhilova et al. under review). MW frequency was calculated using the total number of MW episodes in each task condition divided by the total number of all episodes in each condition (task focus and MW).

|  | **ADHD**  **(N=23)** | **Controls**  **(N=25)** | **Group comparisons** | |
| --- | --- | --- | --- | --- |
|  | **Mean ± SD** | **Mean ± SD** | **d** | **p** |
| **0-back** | 37 ± 7 | 49 ± 10 | **1.35** | 0.001* |
| **1-back** | 169 ± 68 | 224 ± 56 | **0.87** | 0.003* |
| **1s** | 481 ± 166 | 634 ± 83 | **1.16** | 0.000* |
| **2s** | 35 ± 7 | 41 ± 7 | **0.84** | 0.007* |
| **5s** | 37 ± 8 | 46 ± 7 | **1.18** | 0.001* |
| **8s** | 37 ± 7 | 42 ± 8 | **0.65** | 0.027* |
| **MW~** | 88 ± 39 | 39 ± 15 | **1.63** | 0.001* |
| **TOT~** | 90 ± 20 | 158 ± 21 | **2.65** | 0.001* |

**Supplementary Table 2**. Comparisons between ADHD and control groups on the number of artefact-free segments

Abbreviations: ADHD- Attention-deficit/hyperactivity disorder, MW- Mind Wandering episodes, TOT- task focus episodes

Notes: * significant at p≤0.05, **Bold**: d≥.80 indicating large effect size, Italics: d≥.50 indicating a medium effect size, d≥.20 indicating a small effect size. ~This data is based on 21 controls and 21 individuals with ADHD due to the reduced number of MW episodes in controls.

**Supplementary Table 3.** Pearson’s correlations between ERPs in each stimulus type.

|  |  | **Target** | |
| --- | --- | --- | --- |
|  |  | **r** | **p** |
| **Non-target** | P1 | 0.515 | <0.001* |
|  | P3 | 0.778 | <0.001* |

Note: Correlation effect sizes: r≥0.1 small**,** r≥0.3 medium, r≥0.05 large, *p>0.05

**Supplementary Table 4.** Comparisons between groups for all ERPs (P1, P3) in target and non-target trails separately in the Mind Wandering Task

|  |  | **ADHD**  **N=25**  M±SD | **Controls**  **N=23**  M±SD | *d* | p |
| --- | --- | --- | --- | --- | --- |
| **P1** - Target | 1-back | .78±.76 | 1.18±.72 | *0.53* | 0.077 |
|  | 0-back | .64±.65 | 1.38±1.05 | **0.82** | 0.006* |
| **P1** - Non-Target | 1-back | .21±.27 | .70±.90 | **0.71** | 0.015* |
|  | 0-back | .18±.26 | .66±.71 | **0.87** | 0.001* |
| **P3** - Target | 1-back | .40±.36 | .63±.42 | *0.58* | 0.050* |
|  | 0-back | .25±.24 | .42±.34 | *0.56* | 0.062 |
| **P3** - Non-Target | 1-back | .24±.24 | .34±.27 | 0.38 | 0.178 |
|  | 0-back | .06±.09 | .18±.14 | **0.99** | 0 .001* |

Abbreviation: ADHD- Attention-deficit/hyperactivity disorder.

Notes: * significant at p≤0.05, **Bold**: d≥.80 indicating large effect size, Italics: d≥.50 indicating a medium effect size, d≥.20 indicating a small effect size.

| ***Between-group comparisons*** | | | | | | | |
| --- | --- | --- | --- | --- | --- | --- | --- |
| **P3** | | | | **d** | | **p** | |
| ***MWT*** | | | |  | |  | |
|  | | 1-back | | *0.51* | | 0.036* | |
|  | | 0-back | | 0.04 | | 0.135 | |
|  | | MW | | 0.46 | | 0.091 | |
|  | | Task focus | | *0.51* | | 0.024* | |
| ***SAT*** | |  | | **d** | | **p** | |
|  | | 1s | | 0.08 | | 0.231 | |
|  | | 2s | | 0.25 | | 0.913 | |
|  | | 5s | | 0.30 | | 0.730 | |
|  | | 8s | | 0.29 | | 0.826 | |
|  | | MW | | 0.26 | | 0.594 | |
|  | | Task focus | | 0.04 | | 0.983 | |
| ***Within-group comparisons*** | | | | | | | |
|  |  | | **ADHD** | | **Control** | | |
| **P3** |  | | **d** | **p** | **d** | | **p** |
| ***MWT*** | | |  |  |  | |  |
| 1-back vs 0-back | | | *0.65* | 0.007* | **1.00** | | 0.001* |
| MW vs task focus | | | 0.09 | 0.643 | 0.38 | | 0.106 |
| ***SAT*** | | | **d** | **p** | **d** | | **p** |
| 2s vs 5s | | | *0.68* | 0.011* | **0.68** | | 0.008* |
| 2s vs 8s | | | *0.91* | 0.002* | **1.25** | | 0.001* |
| 5s vs 8s | | | 0.46 | 0.071 | *0.67* | | 0.012* |
| MW vs task focus | | | 0.15 | 0.457 | *0.57* | | 0.039* |

**Supplementary Table 5**. Covarying for P1 in the within- and between-group analyses of P3

Abbreviations: MWT- Mind Wandering task, SAT- Sustained Attention Task, MW- Mind Wandering.

Notes: Main and interaction effects for P3 are reported in Table 2 (in the manuscript).

*p<0.05, **Bold**: d≥.80 indicating large effect size, Italics: d≥.50 indicating a medium effect size, d≥.20 indicating a small effect size.

**References**

Kim, S., Liu, Z., Glizer, D., Tannock, R., & Woltering, S. (2014). Adult ADHD and working memory: neural evidence of impaired encoding. *Clinical Neurophysiology*, *125*(8), 1596-1603.
